# QT interval prolongation and mortality in sepsis: a retrospective cohort study from the MIMIC-IV database

**DOI:** 10.1186/s12872-026-05519-z

**Published:** 2026-01-16

**Authors:** Jin-you Zhang, Yang Chen, Ying-xi Zhang, Bao-jun Yang

**Affiliations:** The First Affiliated Hospital of Shantou University Medical College, Shantou, Guangdong China

**Keywords:** Sepsis, QT prolongation, Propensity score matching, Multivariable analysis, Mortality

## Abstract

**Background:**

Sepsis is a leading cause of mortality in patients admitted to the intensive care unit (ICU), and QT prolongation (QTP) is common in critically ill patients. However, the association between QTP and long-term mortality in sepsis remains unexamined.

**Methods:**

This retrospective study included patients meeting the Sepsis-3 criteria upon ICU admission between 2008 and 2019, identified from the Intensive Care Medical Information Mart IV (MIMIC-IV) database. Patients were categorized into two groups based on the presence (QTP group) or absence (non-QTP group) of QTP. Clinical outcomes were compared between patients with and without QTP. We used Kaplan–Meier analysis to compare the 28-day and 1-year all-cause mortality between septic patients with and without QTP. Furthermore, we utilized multivariate regression, propensity score matching (PSM), inverse probability of treatment weighting (IPTW), and a survey-weighted generalized linear model to assess the association of QTP with 28-day and 1-year all-cause mortality in patients with sepsis.

**Results:**

A total of 4,845 patients were enrolled, with 1,424 (29.4%) in the QTP group. Compared with the non-QTP patients, the QTP group had significantly higher 28-day mortality (19.17% vs. 13.15%, *p* < 0.001) and 1-year mortality (33.99% vs. 24.82%, *p* < 0.001). Following PSM, the QTP group exhibited significantly higher mortality at both 28-day mortality (18.81% vs. 15.51%, *p* < 0.05) and 1-year mortality (33.60% vs. 27.64%, *p* < 0.001) compared to the non-QTP group. Patients in the QTP group exhibited increased risk of both 28-day mortality (adjusted OR = 1.34, 95% CI: 1.11–1.61, *p* = 0.002) and 1-year mortality (adjusted OR = 1.40, 95% CI: 1.20–1.63, *p* < 0.001).

**Conclusion:**

The incidence of QTP was significantly elevated in ICU patients with sepsis compared with the general population and QTP was associated with increased risk-adjusted 28-day and 1-year mortality in ICU patients with sepsis.

**Supplementary Information:**

The online version contains supplementary material available at 10.1186/s12872-026-05519-z.

## Introduction

Sepsis, defined as a systemic inflammatory response syndrome (SIRS) resulting from dysregulated host responses to infection [1], is characterized by immune dysfunction, coagulopathy, and subsequent multi-organ failure. Despite advances in medical technology, it remains the leading cause of mortality in intensive care units (ICU) globally. In 2017, an estimated 48.9 million incident cases of sepsis were recorded worldwide and 11.0 million sepsis-related deaths were reported, representing 19.7% of all global deaths [2]. QT prolongation (QTP) is a proarrhythmic status reflecting the increased duration of ventricular activation, which has been identified as a critical risk factor for life-threatening ventricular arrhythmias such as polymorphic ventricular tachycardia (VT) including Torsade de Pointes VT and ventricular fibrillation (VF) and sudden cardiac death in the general population [3, 4]. Additionally, QTP is common in critically ill patients [5, 6], and critically ill patients with QTP have a higher mortality [7, 8]. Critically ill patients are at markedly increased risk of QTP, which is a multifactorial consequence of synergistic pathological triggers. These triggers include electrolyte imbalances, iatrogenic QTP agents, and impaired autonomic regulation [9–11]. QTP is increasingly considered as a direct cardiac sequela of septic myocardial injury [12]. Liu et al found that new-onset QTP occurred in 235/1024 (22.9%) patients with sepsis and patients with QTP had a higher 30-day in-hospital mortality (*p* < 0.001) [13]. However, the association between QTP and long-term mortality in sepsis remains unexamined. Therefore, this retrospective study aims to explore the association between QTP and 28-day and 1-year all-cause mortality in patients with sepsis through the Intensive Care Medical Information Mart IV (MIMIC-IV) database version 2.2 (v2.2) spinning from 2008–2019 and MIMIC-IV-ECG.

## Methods

### Study design and population

This retrospective cohort study extracted data from MIMIC-IV (v2.2) and MIMIC-IV-ECG database. Access to the MIMIC-IV database was granted after the principal investigator completed the required training on the National Institutes of Health platform, including courses on “Study data or Specimens only” and “Conflict of interest” (certification numbers: 62927420 and 62927421). Using the Sepsis-3 criteria, we initially screened 32,970 septic patients. The exclusion criteria were as follows: (1) non-first ICU admissions (*n* = 10,453 excluded); (2) ICU stay duration less than 48 h (*n* = 8,317 excluded); (3) absence of electrocardiogram (ECG) monitoring or ECGs technically unsatisfactory for analysis during ICU stay (*n* = 4,599 excluded); (4) ECG Unreliable for QT interval measurements (e.g., atrial fibrillation (AF), ventricular paced rhythm; *n* = 4,756 excluded). Ultimately, the final cohort comprised 4,845 patients, categorized into a QTP group (*n* = 1,424) and a non-QTP group (*n* = 3,421). To further reduce potential confounding, a 1:1 propensity score matching (PSM) method was used to match the two groups based on 29 baseline covariates (including age, gender, Charlson comorbidity index (CCI), Sequential Organ Failure Assessment (SOFA) score, etc.). The matched cohort comprised 1,393 patients in each group, with a standardized mean difference (SMD) of ≤ 0.1.

### Data collection and variables

The MIMIC-IV-ECG database contains approximately 800,000 standardized 12-lead diagnostic ECGs derived from 160,000 unique hospitalized patients. These ECGs were collected from multiple manufacturers (Burdick/Spacelabs, Philips, and General Electric), with each recording sampled at 500 Hz and spanning a 10-s duration. ECG parameters, including the onset of the QRS, end of the T wave, and R-R interval, were extracted from the MIMIC-IV-ECG database. Corrected QT interval (QTc) was derived using Bazett's formula (QTc = QT/RR^1/2^). The use of Bazett's formula aligns with current standard practice, as reflected in major contemporary guidelines including the 2023 Canadian Cardiovascular Society Clinical Practice Update and the 2022 ESC Guidelines [14, 15]. QTP was defined according to sex-specific criteria: QTc ≥ 470 ms for males and ≥ 480 ms for females [16]. In order to test the robustness of our conclusions to the choice of QT correction formula, we conducted a pre-specified sensitivity analysis using the Framingham linear regression formula (QTc = QT + 0.154 × [1 – RR]) as an alternative method to define QTP, applying the same sex-specific thresholds. Additionally, we collected the following baseline characteristics using structured query language (SQL): (1) Demographic Data: Age and gender; (2) Scoring systems: CCI, Simplified Acute Physiology Score II (SAPS II), and SOFA score; (3) Interventions: Use of mechanical ventilation and vasopressor within the first 24 h of admission; (4) Comorbidities from discharge diagnoses based on International Classification of Diseases and Ninth Revision (ICD-9) codes: Heart failure (HF), chronic kidney disease, liver disease, chronic obstructive pulmonary disease (COPD), coronary artery disease (CAD), and stroke; (5) Vital Signs: Mean arterial pressure (MAP), heart rate, and temperature; (6) Laboratory tests: White blood cell count, hemoglobin, platelet count, serum sodium, serum potassium, serum bicarbonate, serum chloride, serum urea nitrogen (BUN), lactate, pH, partial pressure of oxygen (PO_2_), partial pressure of carbon dioxide (PCO_2_), and creatinine.

### Outcomes

The primary outcomes of our study were the 28-day and 1-year all-cause mortality. Specifically, the 28-day mortality was defined as the incidence of death occurring within 28 days from the time of ICU admission, while the 1-year mortality was defined as the incidence of death occurring within one year of ICU admission. These outcomes were ascertained using patient follow-up records from the MIMIC-IV database.

### Statistical analysis

All statistical analyses were performed using R version 4.4.1 (2024–06–14 ucrt). To address potential bias from missing data, multiple imputation via chained equations (MICE) was performed using the mice package in R. Continuous variables were summarized as mean ± standard deviation (SD) if normally distributed, and as median with interquartile range (IQR) otherwise. Categorical variables were summarized as counts (percentages). Group comparisons utilized chi-square/Fisher’s exact tests for categorical variables and Student’s t-test/Mann–Whitney U test for continuous variables, with test selection guided by normality assumptions and expected frequencies.

To minimize selection bias and confounding effects, PSM was performed using a 1:1 nearest neighbor matching (caliper width = 0.2). The logistic regression was employed for the estimation of patients’ propensity scores for QTP, so that covariate imbalance between the QTP and the non-QTP groups was minimized. An inverse probability of treatment weighting (IPTW) approach, using the estimated propensity scores, was employed to create a weighted cohort designed to address confounding by indication [17]. Additionally, we implemented a survey-weighted generalized linear model adjusted with IPTW to ensure the robustness of our findings. These approaches aimed to address both confounding bias and potential sampling heterogeneity in the MIMIC-IV database. Covariate balance was assessed via SMD, where SMD ≤ 0.1 indicated adequate balance between groups. The Kaplan–Meier survival analysis was used to estimate survival probabilities, and the Tarone-Ware test was applied to determine statistically significant differences between groups.

Furthermore, multivariate logistic regression and multivariate Cox proportional-hazards models were utilized to assess the independent associations between QTP and 28-day and 1-year mortality. In the sensitivity analysis, we applied five association inference models: a multivariate logistic model adjusted with all covariates, a multivariate logistic model adjusted with IPTW, a survey-weighted generalized linear model adjusted with IPTW, a multivariate Cox model adjusted with all covariates, a multivariate cox model adjusted with IPTW. Furthermore, to test the robustness of our primary results to the choice of QT correction formula, we conducted a pre-specified sensitivity analysis. In this analysis, QTc was recalculated using the Framingham linear regression formula, and QTP was redefined using the same sex-specific thresholds. The association between this Framingham-defined QTP and mortality was then reassessed using the same set of five statistical inference models applied to the primary analysis. Hazard ratios (HR) or odds ratios (OR) with 95% confidence intervals (CI) were calculated accordingly. A two-sided *p*-value < 0.05 was considered statistically significant.

## Results

### Baseline characteristics

**Fig. 1:**
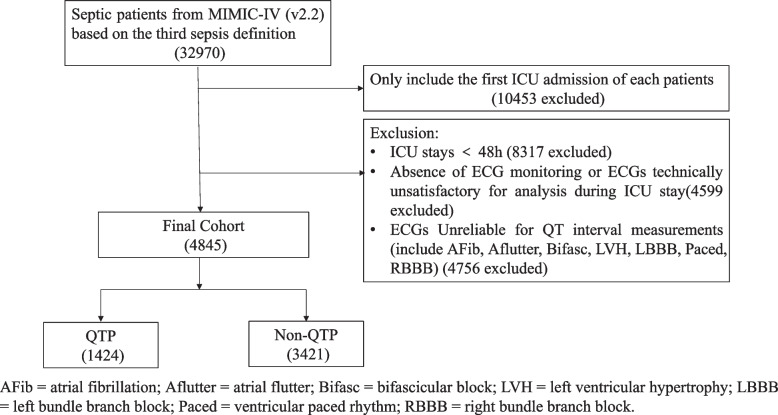
Study flow diagram in the present study

After reviewing data from 32,970 septic patients, a total of 4,845 patients meeting the Sepsis-3 criteria were included in our study, with the selection process detailed in Fig. 1. Among the study cohort, 1,424 patients were categorized as the QTP group and 3,421 as the non-QTP group. The characteristics of the cohort are summarized in Table 1. Patients in the QTP group have greater disease severity as reflected by higher SOFA scores (6.00 [4.00, 9.00] vs. 5.00 [3.00, 8.00]) and SAPS II scores (39.00 [31.00, 49.00] vs. 37.00 [29.00, 46.00]). Patients with QTP had a clinically relevant increase in comorbidity burden, as reflected by a higher CCI (5.00 [3.00, 6.00] vs. 4.00 [2.00, 6.00]). Additionally, the QTP group showed higher prevalence of comorbidities including HF (27.39% vs. 16.81%), chronic kidney disease (18.19% vs. 14.32%), liver disease (13.13% vs. 7.72%), and CAD (31.18% vs. 26.63%).

**Table 1:** Comparisons of baseline characteristics between the original cohort and matched cohort

	Before Matching			After Matching		
	Non-QTP (*N* = 3421)	QTP (*N* = 1424)	SMD	Non-QTP (*N* = 1393)	QTP (*N* = 1393)	SMD
Age, years	62.00 [51.00, 73.00]	63.00 [53.00, 74.00]	0.082	63.00 [53.00, 75.00]	63.00 [53.00, 74.00]	0.005
Gender (Female)	1479 (43.23%)	554 (38.90%)	0.088	563 (40.42%)	548 (39.34%)	0.022
Scoring systems						
CCI	4.00 [2.00, 6.00]	5.00 [3.00, 6.00]	0.168	5.00 [3.00, 7.00]	5.00 [3.00, 6.00]	0.001
SAPSII	37.00 [29.00, 46.00]	39.00 [31.00, 49.00]	0.170	39.00 [31.00, 49.00]	39.00 [31.00, 49.00]	0.011
SOFA	5.00 [3.00, 8.00]	6.00 [4.00, 9.00]	0.250	6.00 [4.00, 9.00]	6.00 [4.00, 9.00]	0.012
Interventions, n (%)						
Mechanical ventilation use (1st 24 h)	2318 (67.76%)	1010 (70.93%)	0.069	1008 (72.36%)	987 (70.85%)	0.033
Vasopressor use (1st 24 h)	1690 (49.40%)	764 (53.65%)	0.085	758 (54.41%)	742 (53.27%)	0.023
Comorbidities, n (%)						
HF	575 (16.81%)	390 (27.39%)	0.257	377 (27.06%)	368 (26.42%)	0.015
Renal	490 (14.32%)	259 (18.19%)	0.105	241 (17.30%)	251 (18.02%)	0.019
Liver	264 (7.72%)	187 (13.13%)	0.178	166 (11.92%)	174 (12.49%)	0.018
COPD	465 (13.59%)	204 (14.33%)	0.021	207 (14.86%)	197 (14.14%)	0.020
CAD	911 (26.63%)	444 (31.18%)	0.101	443 (31.80%)	433 (31.08%)	0.015
Stroke	429 (12.54%)	195 (13.69%)	0.034	202 (14.50%)	191 (13.71%)	0.023
Vital signs						
MAP, mmHg	81.00 [70.00, 92.00]	82.00 [70.00, 95.00]	0.047	81.00 [70.00, 93.00]	82.00 [70.00, 94.00]	0.019
Heart rate	88.00 [77.00, 104.00]	88.00 [76.00, 104.00]	0.001	87.00 [77.00, 103.00]	88.00 [76.00, 104.00]	0.033
Temperature, °C	36.72 [36.39, 37.17]	36.67 [36.33, 37.11]	0.095	36.70 [36.28, 37.11]	36.72 [36.33, 37.11]	0.021
Laboratory tests						
White blood cell, 10^3^/μL	11.80 [8.40, 16.10]	12.10 [8.30, 16.80]	0.023	12.00 [8.60, 16.60]	12.10 [8.30, 16.80]	0.002
Hemoglobin, g/dL	10.70 [9.10, 12.30]	10.40 [8.90, 12.40]	0.039	10.70 [9.10, 12.30]	10.40 [8.90, 12.40]	0.015
Platelet counts, 10^3^/μL	184.00 [129.00, 248.00]	178.00 [122.00, 245.00]	0.071	177.00 [122.00, 248.00]	179.00 [122.00, 247.00]	0.028
Serum sodium, mmol/L	138.00 [135.00, 141.00]	138.00 [135.00, 141.00]	0.017	138.00 [135.00, 141.00]	138.00 [135.00, 141.00]	0.023
Serum potassium, mmol/L	4.20 [3.70, 4.70]	4.10 [3.70, 4.62]	0.078	4.10 [3.70, 4.70]	4.10 [3.70, 4.60]	0.010
Serum bicarbonate, mmol/L	23.00 [20.00, 25.00]	22.00 [19.00, 25.00]	0.149	22.00 [19.00, 25.00]	22.00 [19.00, 25.00]	0.020
Serum chloride, mmol/L	105.00 [101.00, 109.00]	105.00 [100.00, 109.00]	0.064	105.00 [101.00, 109.00]	105.00 [101.00, 109.00]	0.016
Serum urea nitrogen, mg/dL	18.00 [13.00, 28.00]	19.00 [14.00, 31.00]	0.113	19.00 [13.00, 32.00]	19.00 [14.00, 31.00]	0.003
Lactate, mmol/L	1.80 [1.30, 2.70]	2.00 [1.40, 3.10]	0.178	2.00 [1.30, 3.00]	2.00 [1.40, 3.00]	0.010
pH	7.38 [7.30, 7.43]	7.37 [7.31, 7.43]	0.015	7.38 [7.30, 7.43]	7.38 [7.31, 7.43]	0.001
PO_2_, mmHg	143.00 [86.00, 280.00]	139.50 [84.00, 269.25]	0.013	144.00 [83.00, 282.00]	141.00 [84.00, 272.00]	0.003
PCO_2_, mmHg	41.00 [35.00, 47.00]	39.00 [34.00, 45.00]	0.132	39.00 [34.00, 46.00]	39.00 [34.00, 45.00]	0.004
Creatine kinase, mg/dL	0.90 [0.70, 1.40]	1.00 [0.70, 1.60]	0.165	1.00 [0.70, 1.50]	1.00 [0.70, 1.50]	0.005

*Abbreviations*: *QTP QT* prolongation, *CCI* Charlson Comorbidity Index, *SAPSII* Simplified acute physiology score II, *SOFA* Sequential organ failure assessment, *HF* Heart failure, *COPD* chronic obstructive pulmonary disease, *CAD* Coronary artery disease, *MAP* mean arterial pressure

**Fig. 2:**
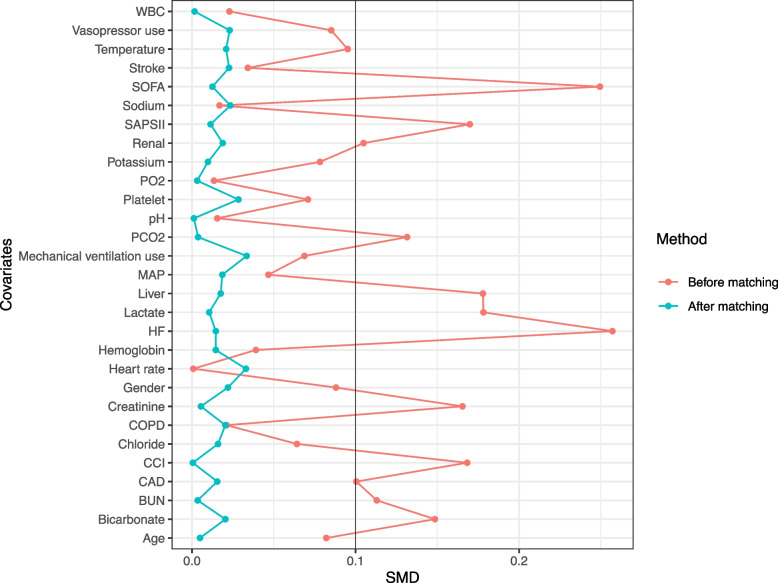
Change in standardized mean difference (SMD) before and after matching of cohort

Before matching, 12 of the 29 covariates had SMDs greater than 0.10; after matching, the SMDs for all 29 covariates were ≤ 0.10, indicating a satisfactory balance between two groups (Fig. 2, Supplementary Table 1). This matching protocol achieved substantial reduction in covariate imbalance, thereby enhancing the reliability of multivariable regression analyses evaluating the independent association between QTP and mortality.

### 28-day survival analysis and sensitivity analyses

**Fig. 3:**
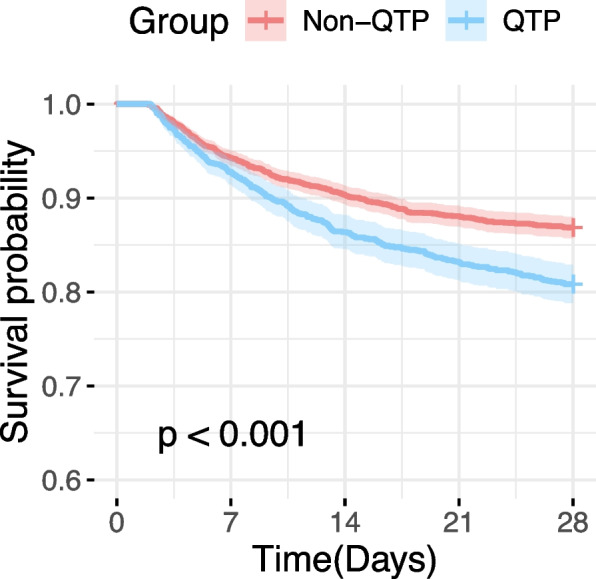
28-day Kaplan–Meier survival curves

Kaplan–Meier survival analysis demonstrated a significantly lower 28-day survival probability in the QTP group compared to the non-QTP group (*p* < 0.001) (Fig. 3). Prior to PSM, the 28-day mortality in the QTP group was significantly higher than that in the non-QTP group (19.17% vs. 13.15%, *p* < 0.001). After PSM, the 28-day mortality in the QTP group remained higher compared to the non-QTP group (18.81% vs. 15.51%, *p* < 0.05) (Table 2). In a multivariate logistic regression analysis adjusted for all covariates, the QTP group exhibited a 34% higher risk of 28-day mortality (OR = 1.34, 95% CI: 1.11–1.61, *p* = 0.002) (Table 3). Furthermore, a multivariate logistic regression model adjusted with IPTW confirmed this result, showing that the 28-day mortality risk remained significantly elevated in the QTP group (OR = 1.32, 95% CI: 1.17–1.49, *p* < 0.001). Additionally, the survey-weighted generalized linear model adjusted with IPTW (OR = 1.32, 95% CI: 1.10–1.59, *p* = 0.003) and the multivariate Cox model adjusted with all covariates (HR = 1.23, 95% CI: 1.05–1.44, *p* = 0.01) further supported this finding.

**Table 2:** Clinical outcomes between the original cohort and matched cohort

	**Before Matching**			**After Matching**		
	**Non-QTP** **(*N* = 3421)**	**QTP** **(*N* = 1424)**	***p* value**	**Non-QTP** **(*N* = 1393)**	**QTP** **(*N* = 1393)**	***p* value**
28-day mortality	450 (13.15%)	273 (19.17%)	< 0.001	216 (15.51%)	262 (18.81%)	< 0.05
1-year mortality	849 (24.82%)	484 (33.99%)	< 0.001	385 (27.64%)	468 (33.60%)	< 0.001

*Abbreviations*: *QTP* QT prolongation

**Table 3:** 28-day mortality analysis with five different models

**Method**	**Adjusted Ratios**	**CI**		***p* value**
		**2.5%**	**97.5%**	
Multivariate logistic model adjusted with all covariates (OR)	1.34	1.11	1.61	0.002
Multivariate logistic model adjusted with IPTW (OR)	1.32	1.17	1.49	< 0.001
Survey-weighted generalized linear model adjusted with IPTW (OR)	1.32	1.10	1.59	0.003
Multivariate Cox model adjusted with all covariates (HR)	1.23	1.05	1.44	0.010
Multivariate Cox model adjusted with IPTW (HR)	1.23	1.05	1.44	0.010

### One-year survival analysis and sensitivity analyses

**Fig. 4:**
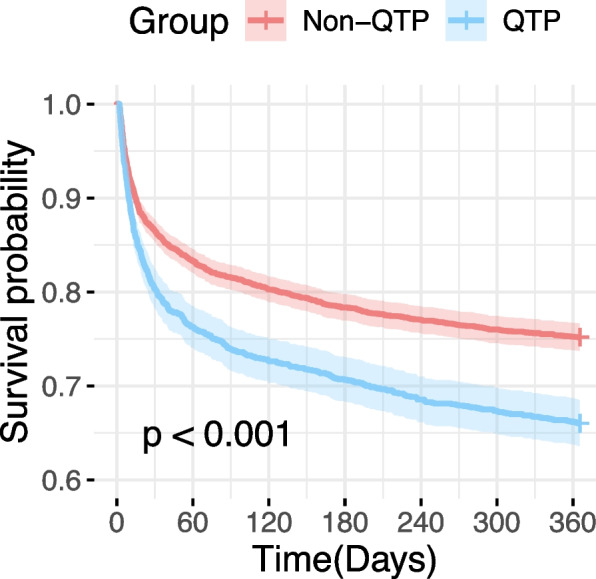
1-year Kaplan–Meier survival curves

For long-term outcomes, Kaplan–Meier survival analysis revealed that the 1-year survival probability was also significantly lower in the QTP group compared to the non-QTP group (*p* < 0.0001) (Fig. 4). Before PSM, the 1-year mortality was higher in the QTP group compared to the non-QTP group (33.99% vs. 24.82%, *p* < 0.001) (Table 2). After PSM, the mortality remained higher in the QTP group than in the non-QTP group (33.60% vs. 27.64%, *p* < 0.001).

In a multivariate logistic regression analysis adjusted for all covariates, the QTP group was associated with increased risk of 1-year mortality (OR = 1.40, 95% CI: 1.20–1.63, *p* < 0.001) (Table 4). This result was further supported by a multivariate logistic regression model adjusted with IPTW, which demonstrated that the QTP group had significantly elevated odds of 1-year mortality (OR = 1.34, 95% CI: 1.22–1.48, *p* < 0.001). Consistent results were also observed in the survey-weighted generalized linear model adjusted with IPTW (OR = 1.34, 95% CI: 1.15–1.57, *p* < 0.001) and the multivariate Cox model adjusted with all covariates (HR = 1.23, 95% CI: 1.10–1.38, *p* < 0.001).

**Table 4:** 1-year mortality analysis with five different models

**Method**	**Adjusted Ratios**	**CI**		***p* value**
		**2.5%**	**97.5%**	
Multivariate logistic model adjusted with all covariates (OR)	1.40	1.20	1.63	< 0.001
Multivariate logistic model adjusted with IPTW (OR)	1.34	1.22	1.48	< 0.001
Survey-weighted generalized linear model adjusted with IPTW (OR)	1.34	1.15	1.57	< 0.001
Multivariate Cox model adjusted with all covariates (HR)	1.23	1.10	1.38	< 0.001
Multivariate Cox model adjusted with IPTW (HR)	1.22	1.08	1.37	0.001

### Sensitivity analysis using the Framingham correction formula

To test the robustness of our primary results to the methodological choice of QT correction, we conducted a sensitivity analysis redefining QTP using the Framingham linear regression formula. The full results are presented in Supplementary Tables 12 (28-day mortality) and 13 (1-year mortality). In brief, the direction and magnitude of the association were consistent with our primary analysis. For 28-day mortality, Framingham-defined QTP was associated with increased risk across all five analytical models, with point estimates (HRs/ORs) consistently greater than 1.0. This association reached statistical significance (*p* < 0.05) in four of the five models; in the multivariate Cox model adjusted with all covariates, the association showed a non-significant trend (HR = 1.22, 95% CI: 0.97–1.52, *p* = 0.08). For 1-year mortality, the association was statistically significant in all five models (all *p* < 0.01). These results confirm that the independent association between QTP and increased mortality in sepsis is robust.

## Discussion

Utilizing a large cohort from the MIMIC-IV database, this study systematically demonstrated an independent association between QTP and both short-term and long-term mortality in ICU patients with sepsis. The independent prognostic value of QTP was consistently demonstrated across multiple statistical models, including multivariate logistic model adjusted with all covariates, multivariate logistic model adjusted with IPTW, survey-weighted generalized linear model adjusted with IPTW, multivariate Cox model adjusted with all covariates, as well as multivariate cox model adjusted with IPTW. Importantly, the association between QTP and mortality proved consistent when using two different QT correction methods. Our Conclusion based on Bazett’s formula were supported by a sensitivity analysis using the Framingham formula (Supplementary Tables 12 and 13). Although statistical significance was borderline in one model for 28-day mortality, the uniform trend (all risk ratios > 1) reinforces the credibility of the observed association between QTP and mortality. 

QTP can be either congenital, inherited as an autosomal dominant variant, or acquired [18–20]. In our study, QTP was not explicitly classified as congenital or acquired. The QT interval, measured from the onset of the QRS complex to the end of the T wave, is generally associated with abnormal cardiac electrical activity, potentially increasing the risk of malignant arrhythmias and consequently affecting patient outcome [6].

To ensure accurate QT interval measurement, ECGs with abnormalities that confound QT interval assessment were excluded, including AF, bundle branch block, ventricular-paced rhythm, etc. [3, 21, 22]. Different formulas exist for QTc calculation, each with its own limitations. The accuracy of Bazett's formula is considered optimal for heart rates between 60 and 100 beats per minute. Given its widespread use in clinical practice, we employed Bazett's formula for QTc calculation in our study. Moreover, some consensus recommendations suggest upper limits of normal QTc as 470 ms for males and 480 ms for females [16, 23]. Therefore, we adopted these values as the criteria for defining QTP in our study.

In our study, the incidence of QTP among ICU patients with sepsis was 29.4%. This rate is higher than the 22.9% incidence reported by Liu et al., which may be attributed to their exclusion of patients with a history of congenital or other acquired long QT syndrome, regular intake of QT interval-affecting medications, and administration of QT-influencing antibiotics prior to ECG testing in their study [13]. Additionally, compared to the reported incidence of acquired QTP of 7.82% in the emergency department settings [24], and the estimated prevalence of Congenital Long QT Syndrome (LQTS) ranging from 1/5,000 [25] to 1/20,000 [26] (with estimates generally around 1/10,000 [20]), our study demonstrates a significantly higher occurrence of QTP in septic ICU patients than typically observed in the general population.

Our study indicates that QTP is independently associated with adverse outcomes in sepsis patients. The potential mechanism may be multifactorial, involving both chronic baseline susceptibility and acute-phase triggers of sepsis. Our study observed a higher prevalence of comorbid HF (27.39% vs. 16.81%), chronic kidney disease (18.19% vs. 14.32%), liver disease (13.13% vs. 7.72%), and CAD (31.18% vs. 26.63%) in the QTP group. This aligns with established pathophysiological mechanisms. In previous studies, Dahl salt-sensitive rats were fed a high-salt diet to induce Heart failure with preserved ejection fraction. These rats exhibited delayed repolarization from downregulation of potassium currents and QTP, as reported by Jae Hyung et al. in Circulation [27]. Further contributing to QTP susceptibility, polymorphisms in the angiotensin-converting enzyme and angiotensin II type 1 receptor genes contribute additively to QTP, which is prevalent in the majority of end-stage renal disease patients [28]. Clinically, liver transplantation leads to significant improvement in prolonged QTc interval in patients with end-stage liver disease [29], while previous study indicated QTP has been associated with CAD [30]. Building upon this chronic susceptible substrate, the acute phase of sepsis can directly induce or exacerbate QTP through several interconnected pathways. Firstly, sepsis-related myocardial injury is known to precipitate QTP [12]. Secondly, during sepsis, the release of a vast array of inflammatory mediators—particularly cytokines such as interleukin-1, interleukin-6—prolongs ventricular repolarization. Mechanistically, interleukin-1 inhibits the expression of the K_ir_2.1 channel, while interleukin-6 activates the interleukin-6 receptor and Janus kinase signaling pathway to suppress the rapid delayed rectifier potassium current. thereby establishing a direct electrophysiological link between systemic inflammation and QTP [31, 32]. Notably, elevated levels of interleukin-6 have been identified as an independent risk factor for QTP [33]. Thirdly, electrolyte disturbances commonly observed in sepsis—such as hypokalemia, hypomagnesemia, and hypocalcemia—contribute significantly to QTP. Hypokalemia reduces the rapid delayed rectifier potassium current, while hypomagnesemia and hypocalcemia are well-established factors that facilitate QTP [14, 34]. Fourthly, pharmacological management of sepsis introduces additional risk, as several essential drugs, notably fluoroquinolone and macrolide antibiotics, possess inherent QT-prolonging potential [35, 36]. Finally, this drug-induced risk is further amplified by sepsis-induced organ dysfunction: acute kidney and liver injuries impair the clearance and metabolism of these medications, leading to their accumulation and increasing the pro-arrhythmic burden on an already vulnerable myocardium. These acute factors interact synergistically with preexisting chronic susceptibilities, which can culminate in clinically significant QTP.

To contextualize the prognostic value of QTP, we compared its effect size with established cardiac biomarkers via published meta-analyses. In sepsis, elevated troponin (adjusted OR = 1.92, 95% CI: 1.35–2.74) and NT-proBNP (adjusted OR = 1.36, 95% CI: 1.20–1.54) are robust mortality predictors [37, 38]. Our study found adjusted ORs for QTP of 1.34 (28-day) and 1.40 (1-year), indicating a similar predictive strength to NT-proBNP. This suggests QTP provides complementary prognostic information, potentially reflecting distinct electrophysiological risk versus direct myocardial injury. Crucially, QTP offers unique bedside accessibility from the routine admission ECG.

Collectively, these observations suggest that QTP may serve as an electrophysiological indicator of the cumulative burden or severity of these high-risk comorbidities. Given that these severe comorbidities themselves increase 28-day mortality in septic patients, a critical question arises: to what extent is the observed association between QTP and both 28-day mortality and 1-year mortality attributable to the effect of QTP itself, versus reflecting the higher mortality risk conferred by these underlying comorbidities? Therefore, our study employed PSM to adjust for confounding factors. After PSM, the SMD for comorbidities between the QTP and the non-QTP groups were all ≤ 0.1. Crucially, even after PSM, the QTP group still exhibited significantly higher 28-day mortality (*p* < 0.05) and 1-year mortality (*p* < 0.001) compared to the non-QTP group. Furthermore, even after adjusting for 29 covariates, including HF, liver disease, kidney disease, and CAD, analyses of both 28-day mortality and 1-year mortality using five different models consistently demonstrated that QTP was associated with a risk ratio greater than 1, with p-values less than 0.05. This indicates that QTP may possess independent prognostic value beyond these specific comorbidities.

QTP may contribute directly or indirectly to increased mortality risk in sepsis through at least three interconnected pathways. First, it carries an inherent pro-arrhythmic risk, predisposing to *torsades de pointes*—a polymorphic ventricular tachycardia that can degenerate into ventricular fibrillation and cause sudden cardiac death [39, 40]. Second, QTP serves as an indicator of overall disease severity [41]. Third, the presence of QTP may constrain therapeutic options by contraindicating the use of otherwise effective but QT-prolonging medications (e.g., certain anti-infectives), potentially compromising the intensity of care. As QTP is readily assessable via routine ECG, it represents a valuable bedside indicator for prognostic evaluation in ICU patients with sepsis. Given its independent prognostic value and ease of measurement, QTP should be routinely assessed on admission ECGs in septic ICU patients. A finding of QTP should prompt heightened vigilance for arrhythmias, more frequent electrolyte monitoring (particularly potassium and magnesium), and a review of medications for QT-prolonging effects, even in the absence of overt arrhythmia. Such measures could contribute to early risk stratification and timely intervention in this high-risk population. Given this prognostic value and ease of assessment, several clinical practice recommendations can be made: Firstly, routine 12-lead ECG with QTc calculation should be considered for all sepsis patients upon ICU admission. For those identified with QTP, increased ECG monitoring frequency is advisable, particularly during clinical deterioration or upon initiation of new medications. Secondly, a thorough medication review is essential for patients with QTP. Clinicians should exercise heightened vigilance and, whenever possible, avoid agents known to prolong the QT interval. When use of such drugs is unavoidable, close ECG monitoring and prompt correction of modifiable risk factors (e.g., hypokalemia) are imperative. Finally, QTP can be utilized to refine risk stratification and aid in prognosis communication with families. Future research could explore whether incorporating QTP into existing sepsis prognostic scores (e.g., SOFA) enhances their predictive accuracy.

Our study has several limitations. First, as a single-center retrospective study, selection and information biases may limit the generalizability of the results. Second, we did not include information on medications that affect the QT interval. Third, our study did not differentiate between acquired and congenital QTP, which may contribute to heterogeneity in the findings. Fourth, although we adjusted for the presence of major comorbidities using ICD codes, our data did not capture their severity or functional status (e.g., New York Heart Association class for heart failure, stages of chronic kidney disease, or left ventricular ejection fraction), which could represent residual confounding. Fifth, this study did not directly compare the prognostic performance of QTP with that of established cardiac biomarkers (e.g., troponin, BNP/NT-proBNP) due to the prohibitively high rate of missing laboratory data in the MIMIC-IV database.

## Conclusion

The incidence of QTP was significantly elevated in ICU patients with sepsis compared with the general population. Our study demonstrated that QTP was associated with increased risk-adjusted 28-day and 1-year mortality in ICU patients with sepsis. Considering the ease of obtaining ECG, the QTP may serve as a simple bedside indicator for prognostic evaluation in ICU patients with sepsis.

## Supplementary Information

Supplementary Material 1.

## Data Availability

The datasets analyzed during the current study are available in the MIMIC database.

## Abbreviations

AF: Atrial fibrillation
BUN: Serum urea nitrogen
CAD: Coronary artery disease
CCI: Charlson Comorbidity Index
CI: Confidence intervals
COPD: Chronic obstructive pulmonary disease
ECG: Electrocardiogram
HF: Heart failure
HR: Hazard ratios
IQR: Interquartile range
ICD-9: International Classification of Diseases and Ninth Revision
IPTW: Inverse probability of treatment weighting
ICU: Intensive care unit
LQTS: Congenital Long QT Syndrome
MAP: Mean arterial pressure
MICE: Multiple imputation via chained equations
MIMIC-IV: Intensive Care Medical Information Mart IV
OR: Odds ratios
PCO_2_: Partial pressure of carbon dioxide
PO_2_: Partial pressure of oxygen
PSM: Propensity score matching
QTP: QT interval prolongation
QTc: QT interval corrected for heart rate
SD: Standard deviation
SIRS: Systemic inflammatory response syndrome
SOFA: Sequential Organ Failure Assessment
SQL: Structured query language
SAPS II: Simplified Acute Physiology Score II
SMD: Standardized mean difference
VT: Ventricular tachycardia
VF: Ventricular fibrillation
