# Supplementary material for: QT interval prolongation and mortality in sepsis: a retrospective cohort study from the MIMIC-IV database
Source: BMC Cardiovasc Disord. 2026 Jan 16;26:67. doi: 10.1186/s12872-026-05519-z (PMC12825248; doi:10.1186/s12872-026-05519-z)
Supplement: Supplementary file 1 — Supplementary Material 1. [file 12872_2026_5519_MOESM1_ESM.docx]

Supplementary Table 1. Standardized mean difference (SMD) of covariates before and after propensity score matching of cohort

| Characteristic | Before matcing | After matcing |
| --- | --- | --- |
| SMD ≤ 0.1 | 17 | 29 |
| SMD > 0.1 | 12 | 0 |
| Total number of covariates | 29 | 29 |

Supplementary Table 2. Multivariate logistic model adjusted with all covariates for 28-day mortality of original cohort

| **Characteristic** | **OR**^1^ | **95% CI**^1^ | **p-value** |
| --- | --- | --- | --- |
| Group |  |  |  |
| Non-QTP | 1.00 | Reference |  |
| QTP | 1.34 | 1.11, 1.61 | 0.002 |
| Age | 1.01 | 1.00, 1.02 | 0.04 |
| Gender |  |  |  |
| Female | 1.00 | Reference |  |
| Male | 1.09 | 0.91, 1.31 | 0.36 |
| CCI | 1.16 | 1.11, 1.21 | <0.001 |
| SAPSII | 1.01 | 1.01, 1.02 | 0.002 |
| SOFA | 1.12 | 1.07, 1.17 | <0.001 |
| Mechanical ventilation use |  |  |  |
| NO | 1.00 | Reference |  |
| YES | 0.85 | 0.69, 1.06 | 0.15 |
| Vasopressor use |  |  |  |
| NO | 1.00 | Reference |  |
| YES | 0.79 | 0.63, 0.98 | 0.03 |
| HF |  |  |  |
| NO | 1.00 | Reference |  |
| YES | 0.86 | 0.69, 1.08 | 0.19 |
| Renal |  |  |  |
| NO | 1.00 | Reference |  |
| YES | 0.77 | 0.58, 1.00 | 0.05 |
| Liver |  |  |  |
| NO | 1.00 | Reference |  |
| YES | 1.29 | 0.97, 1.71 | 0.08 |
| COPD |  |  |  |
| NO | 1.00 | Reference |  |
| YES | 1.09 | 0.85, 1.38 | 0.5 |
| CAD |  |  |  |
| NO | 1.00 | Reference |  |
| YES | 0.63 | 0.50, 0.79 | <0.001 |
| Stroke |  |  |  |
| NO | 1.00 | Reference |  |
| YES | 1.43 | 1.12, 1.83 | 0.004 |
| MAP | 1.00 | 1.00, 1.01 | 0.8 |
| Heart rate | 1.01 | 1.00, 1.01 | 0.007 |
| Temperature | 0.84 | 0.76, 0.92 | <0.001 |
| WBC | 1.00 | 1.00, 1.01 | 0.26 |
| Hemoglobin | 1.01 | 0.97, 1.05 | 0.77 |
| Platelet | 1.00 | 1.00, 1.00 | 0.002 |
| Sodium | 1.05 | 1.02, 1.07 | <0.001 |
| Potassium | 0.93 | 0.83, 1.04 | 0.21 |
| Bicarbonate | 0.99 | 0.96, 1.01 | 0.33 |
| Chloride | 0.95 | 0.93, 0.97 | <0.001 |
| BUN | 1.01 | 1.00, 1.01 | 0.02 |
| Lactate | 1.00 | 0.95, 1.05 | 0.89 |
| pH | 0.47 | 0.12, 1.85 | 0.28 |
| PO2 | 1.00 | 1.00, 1.00 | <0.001 |
| PCO2 | 0.99 | 0.98, 1.00 | 0.04 |
| Creatinine | 0.86 | 0.79, 0.94 | 0.001 |
| ^1^OR = Odds Ratio, CI = Confidence Interval | | | |

Supplementary Table 3. Multivariate logistic model adjusted with IPTW for 28-day mortality of cohort

| **Characteristic** | **OR**^1^ | **95% CI**^1^ | **p-value** |
| --- | --- | --- | --- |
| Group |  |  |  |
| Non-QTP | 1.00 | Reference |  |
| QTP | 1.32 | 1.17, 1.49 | <0.001 |
| Age | 1.01 | 1.01, 1.02 | <0.001 |
| Gender |  |  |  |
| Female | 1.00 | Reference |  |
| Male | 1.16 | 1.02, 1.32 | 0.02 |
| CCI | 1.13 | 1.10, 1.17 | <0.001 |
| SAPSII | 1.01 | 1.00, 1.02 | <0.001 |
| SOFA | 1.14 | 1.11, 1.18 | <0.001 |
| Mechanical ventilation use |  |  |  |
| NO | 1.00 | Reference |  |
| YES | 0.87 | 0.75, 1.00 | 0.06 |
| Vasopressor use |  |  |  |
| NO | 1.00 | Reference |  |
| YES | 0.76 | 0.65, 0.89 | <0.001 |
| HF |  |  |  |
| NO | 1.00 | Reference |  |
| YES | 0.82 | 0.70, 0.96 | 0.01 |
| Renal |  |  |  |
| NO | 1.00 | Reference |  |
| YES | 0.75 | 0.62, 0.90 | 0.003 |
| Liver |  |  |  |
| NO | 1.00 | Reference |  |
| YES | 1.28 | 1.05, 1.57 | 0.01 |
| COPD |  |  |  |
| NO | 1.00 | Reference |  |
| YES | 1.26 | 1.07, 1.49 | 0.007 |
| CAD |  |  |  |
| NO | 1.00 | Reference |  |
| YES | 0.64 | 0.55, 0.75 | <0.001 |
| Stroke |  |  |  |
| NO | 1.00 | Reference |  |
| YES | 1.45 | 1.22, 1.71 | <0.001 |
| MAP | 1.00 | 1.00, 1.00 | 0.36 |
| Heart rate | 1.00 | 1.00, 1.01 | 0.005 |
| Temperature | 0.83 | 0.77, 0.88 | <0.001 |
| WBC | 1.00 | 1.00, 1.01 | 0.13 |
| Hemoglobin | 1.03 | 1.00, 1.06 | 0.03 |
| Platelet | 1.00 | 1.00, 1.00 | <0.001 |
| Sodium | 1.04 | 1.02, 1.06 | <0.001 |
| Potassium | 0.94 | 0.87, 1.02 | 0.13 |
| Bicarbonate | 0.98 | 0.96, 1.00 | 0.02 |
| Chloride | 0.95 | 0.94, 0.97 | <0.001 |
| BUN | 1.01 | 1.00, 1.01 | <0.001 |
| Lactate | 0.99 | 0.95, 1.02 | 0.52 |
| pH | 0.60 | 0.23, 1.55 | 0.29 |
| PO2 | 1.00 | 1.00, 1.00 | <0.001 |
| PCO2 | 0.99 | 0.98, 0.99 | <0.001 |
| Creatinine | 0.85 | 0.79, 0.90 | <0.001 |
| ^1^OR = Odds Ratio, CI = Confidence Interval | | | |
|  | | | |

Supplementary Table 4. Survey-weighted generalised linear model adjusted with IPTW for 28-day mortality of cohort

| **Characteristic** | **OR**^1^ | **95% CI**^1^ | **p-value** |
| --- | --- | --- | --- |
| Group |  |  |  |
| Non-QTP | 1.00 | Reference |  |
| QTP | 1.32 | 1.10, 1.59 | 0.003 |
| Age | 1.01 | 1.00, 1.02 | 0.01 |
| Gender |  |  |  |
| Female | 1.00 | Reference |  |
| Male | 1.16 | 0.95, 1.42 | 0.16 |
| CCI | 1.13 | 1.08, 1.19 | <0.001 |
| SAPSII | 1.01 | 1.00, 1.02 | 0.04 |
| SOFA | 1.14 | 1.09, 1.20 | <0.001 |
| Mechanical ventilation use |  |  |  |
| NO | 1.00 | Reference |  |
| YES | 0.87 | 0.68, 1.10 | 0.23 |
| Vasopressor use |  |  |  |
| NO | 1.00 | Reference |  |
| YES | 0.76 | 0.59, 0.98 | 0.03 |
| HF |  |  |  |
| NO | 1.00 | Reference |  |
| YES | 0.82 | 0.64, 1.04 | 0.1 |
| Renal |  |  |  |
| NO | 1.00 | Reference |  |
| YES | 0.75 | 0.56, 1.01 | 0.06 |
| Liver |  |  |  |
| NO | 1.00 | Reference |  |
| YES | 1.28 | 0.96, 1.72 | 0.1 |
| COPD |  |  |  |
| NO | 1.00 | Reference |  |
| YES | 1.26 | 0.96, 1.65 | 0.09 |
| CAD |  |  |  |
| NO | 1.00 | Reference |  |
| YES | 0.64 | 0.49, 0.83 | <0.001 |
| Stroke |  |  |  |
| NO | 1.00 | Reference |  |
| YES | 1.45 | 1.10, 1.90 | 0.008 |
| MAP | 1.00 | 1.00, 1.01 | 0.57 |
| Heart rate | 1.00 | 1.00, 1.01 | 0.08 |
| Temperature | 0.83 | 0.73, 0.93 | 0.001 |
| WBC | 1.00 | 1.00, 1.01 | 0.38 |
| Hemoglobin | 1.03 | 0.99, 1.08 | 0.17 |
| Platelet | 1.00 | 1.00, 1.00 | 0.001 |
| Sodium | 1.04 | 1.01, 1.07 | 0.005 |
| Potassium | 0.94 | 0.83, 1.07 | 0.35 |
| Bicarbonate | 0.98 | 0.94, 1.01 | 0.15 |
| Chloride | 0.95 | 0.93, 0.98 | <0.001 |
| BUN | 1.01 | 1.00, 1.01 | 0.03 |
| Lactate | 0.99 | 0.93, 1.05 | 0.69 |
| pH | 0.60 | 0.11, 3.16 | 0.55 |
| PO2 | 1.00 | 1.00, 1.00 | <0.001 |
| PCO2 | 0.99 | 0.97, 1.00 | 0.07 |
| Creatinine | 0.85 | 0.77, 0.93 | <0.001 |
| ^1^OR = Odds Ratio, CI = Confidence Interval | | | |

Supplementary Table 5. Multivariate Cox model adjusted with all covariates for 28-day mortality of original cohort

| **Characteristic** | **HR**^1^ | **95% CI**^1^ | **p-value** |
| --- | --- | --- | --- |
| Group |  |  |  |
| Non-QTP | 1.00 | Reference |  |
| QTP | 1.23 | 1.05, 1.44 | 0.01 |
| Age | 1.01 | 1.00, 1.01 | 0.03 |
| Gender |  |  |  |
| Female | 1.00 | Reference |  |
| Male | 1.07 | 0.91, 1.25 | 0.4 |
| CCI | 1.13 | 1.09, 1.17 | <0.001 |
| SAPSII | 1.01 | 1.00, 1.02 | 0.003 |
| SOFA | 1.10 | 1.07, 1.14 | <0.001 |
| Mechanical ventilation use |  |  |  |
| NO | 1.00 | Reference |  |
| YES | 0.86 | 0.71, 1.04 | 0.11 |
| Vasopressor use |  |  |  |
| NO | 1.00 | Reference |  |
| YES | 0.81 | 0.67, 0.98 | 0.03 |
| HF |  |  |  |
| NO | 1.00 | Reference |  |
| YES | 0.91 | 0.75, 1.10 | 0.34 |
| Renal |  |  |  |
| NO | 1.00 | Reference |  |
| YES | 0.80 | 0.64, 1.00 | 0.05 |
| Liver |  |  |  |
| NO | 1.00 | Reference |  |
| YES | 1.23 | 0.97, 1.55 | 0.09 |
| COPD |  |  |  |
| NO | 1.00 | Reference |  |
| YES | 1.09 | 0.89, 1.33 | 0.42 |
| CAD |  |  |  |
| NO | 1.00 | Reference |  |
| YES | 0.67 | 0.55, 0.83 | <0.001 |
| Stroke |  |  |  |
| NO | 1.00 | Reference |  |
| YES | 1.35 | 1.10, 1.66 | 0.005 |
| MAP | 1.00 | 1.00, 1.00 | 0.64 |
| Heart rate | 1.01 | 1.00, 1.01 | 0.005 |
| Temperature | 0.86 | 0.79, 0.93 | <0.001 |
| WBC | 1.00 | 1.00, 1.01 | 0.32 |
| Hemoglobin | 1.02 | 0.98, 1.05 | 0.38 |
| Platelet | 1.00 | 1.00, 1.00 | 0.002 |
| Sodium | 1.04 | 1.02, 1.06 | <0.001 |
| Potassium | 0.95 | 0.86, 1.04 | 0.27 |
| Bicarbonate | 0.99 | 0.96, 1.01 | 0.27 |
| Chloride | 0.96 | 0.94, 0.97 | <0.001 |
| BUN | 1.01 | 1.00, 1.01 | 0.02 |
| Lactate | 0.99 | 0.95, 1.03 | 0.7 |
| pH | 0.54 | 0.18, 1.62 | 0.27 |
| PO2 | 1.00 | 1.00, 1.00 | 0.001 |
| PCO2 | 0.99 | 0.98, 1.00 | 0.06 |
| Creatinine | 0.89 | 0.83, 0.96 | 0.002 |
| ^1^HR = Hazard Ratio, CI = Confidence Interval | | | |

Supplementary Table 6. Multivariate Cox model adjusted with IPTW for 28-day mortality of cohort

| **Characteristic** | **HR**^1^ | **95% CI**^1^ | **p-value** |
| --- | --- | --- | --- |
| Group |  |  |  |
| Non-QTP | 1.00 | Reference |  |
| QTP | 1.23 | 1.05, 1.44 | 0.01 |
| Age | 1.01 | 1.00, 1.02 | 0.01 |
| Gender |  |  |  |
| Female | 1.00 | Reference |  |
| Male | 1.12 | 0.94, 1.34 | 0.19 |
| CCI | 1.11 | 1.07, 1.15 | <0.001 |
| SAPSII | 1.01 | 1.00, 1.02 | 0.06 |
| SOFA | 1.13 | 1.08, 1.17 | <0.001 |
| Mechanical ventilation use |  |  |  |
| NO | 1.00 | Reference |  |
| YES | 0.88 | 0.72, 1.08 | 0.22 |
| Vasopressor use |  |  |  |
| NO | 1.00 | Reference |  |
| YES | 0.79 | 0.64, 0.98 | 0.04 |
| HF |  |  |  |
| NO | 1.00 | Reference |  |
| YES | 0.87 | 0.70, 1.07 | 0.18 |
| Renal |  |  |  |
| NO | 1.00 | Reference |  |
| YES | 0.77 | 0.61, 0.99 | 0.04 |
| Liver |  |  |  |
| NO | 1.00 | Reference |  |
| YES | 1.22 | 0.96, 1.55 | 0.11 |
| COPD |  |  |  |
| NO | 1.00 | Reference |  |
| YES | 1.23 | 0.98, 1.54 | 0.08 |
| CAD |  |  |  |
| NO | 1.00 | Reference |  |
| YES | 0.68 | 0.54, 0.86 | 0.001 |
| Stroke |  |  |  |
| NO | 1.00 | Reference |  |
| YES | 1.35 | 1.08, 1.69 | 0.008 |
| MAP | 1.00 | 1.00, 1.01 | 0.42 |
| Heart rate | 1.00 | 1.00, 1.01 | 0.06 |
| Temperature | 0.86 | 0.78, 0.94 | 0.002 |
| WBC | 1.00 | 1.00, 1.01 | 0.35 |
| Hemoglobin | 1.04 | 1.00, 1.08 | 0.05 |
| Platelet | 1.00 | 1.00, 1.00 | 0.002 |
| Sodium | 1.03 | 1.01, 1.06 | 0.005 |
| Potassium | 0.96 | 0.86, 1.07 | 0.46 |
| Bicarbonate | 0.98 | 0.95, 1.01 | 0.13 |
| Chloride | 0.96 | 0.94, 0.98 | <0.001 |
| BUN | 1.01 | 1.00, 1.01 | 0.02 |
| Lactate | 0.99 | 0.94, 1.04 | 0.58 |
| pH | 0.73 | 0.20, 2.72 | 0.64 |
| PO2 | 1.00 | 1.00, 1.00 | 0.002 |
| PCO2 | 0.99 | 0.98, 1.00 | 0.1 |
| Creatinine | 0.88 | 0.81, 0.95 | 0.001 |
| ^1^HR = Hazard Ratio, CI = Confidence Interval | | | |

Supplementary Table 7. Multivariate logistic model adjusted with all covariates for 1-year mortality of original cohort

| **Characteristic** | **OR**^1^ | **95% CI**^1^ | **p-value** |
| --- | --- | --- | --- |
| Group |  |  |  |
| Non-QTP | 1.00 | Reference |  |
| QTP | 1.40 | 1.20, 1.63 | <0.001 |
| Age | 1.00 | 1.00, 1.01 | 0.17 |
| Gender |  |  |  |
| Female | 1.00 | Reference |  |
| Male | 1.06 | 0.91, 1.24 | 0.42 |
| CCI | 1.26 | 1.22, 1.31 | <0.001 |
| SAPSII | 1.02 | 1.02, 1.03 | <0.001 |
| SOFA | 1.07 | 1.03, 1.11 | <0.001 |
| Mechanical ventilation use |  |  |  |
| NO | 1.00 | Reference |  |
| YES | 0.80 | 0.67, 0.95 | 0.01 |
| Vasopressor use |  |  |  |
| NO | 1.00 | Reference |  |
| YES | 0.79 | 0.66, 0.94 | 0.01 |
| HF |  |  |  |
| NO | 1.00 | Reference |  |
| YES | 0.82 | 0.68, 0.99 | 0.04 |
| Renal |  |  |  |
| NO | 1.00 | Reference |  |
| YES | 0.63 | 0.50, 0.79 | <0.001 |
| Liver |  |  |  |
| NO | 1.00 | Reference |  |
| YES | 1.44 | 1.13, 1.85 | 0.004 |
| COPD |  |  |  |
| NO | 1.00 | Reference |  |
| YES | 1.31 | 1.07, 1.60 | 0.008 |
| CAD |  |  |  |
| NO | 1.00 | Reference |  |
| YES | 0.59 | 0.49, 0.71 | <0.001 |
| Stroke |  |  |  |
| NO | 1.00 | Reference |  |
| YES | 1.29 | 1.04, 1.59 | 0.02 |
| MAP | 1.00 | 1.00, 1.01 | 0.36 |
| Heart rate | 1.00 | 1.00, 1.01 | 0.01 |
| Temperature | 0.83 | 0.76, 0.90 | <0.001 |
| WBC | 1.01 | 1.00, 1.01 | 0.12 |
| Hemoglobin | 0.96 | 0.92, 0.99 | 0.01 |
| Platelet | 1.00 | 1.00, 1.00 | 0.002 |
| Sodium | 1.05 | 1.03, 1.07 | <0.001 |
| Potassium | 0.88 | 0.80, 0.97 | 0.01 |
| Bicarbonate | 0.99 | 0.97, 1.02 | 0.44 |
| Chloride | 0.95 | 0.93, 0.96 | <0.001 |
| BUN | 1.01 | 1.00, 1.01 | 0.04 |
| Lactate | 0.97 | 0.92, 1.01 | 0.16 |
| pH | 0.36 | 0.11, 1.19 | 0.09 |
| PO2 | 1.00 | 1.00, 1.00 | <0.001 |
| PCO2 | 0.99 | 0.98, 1.00 | 0.02 |
| Creatinine | 0.90 | 0.83, 0.96 | 0.003 |
| ^1^OR = Odds Ratio, CI = Confidence Interval | | | |

Supplementary Table 8. Multivariate logistic model adjusted with IPTW for 1-year mortality of cohort

| **Characteristic** | **OR**^1^ | **95% CI**^1^ | **p-value** |
| --- | --- | --- | --- |
| Group |  |  |  |
| Non-QTP | 1.00 | Reference |  |
| QTP | 1.34 | 1.22, 1.48 | <0.001 |
| Age | 1.01 | 1.00, 1.01 | 0.002 |
| Gender |  |  |  |
| Female | 1.00 | Reference |  |
| Male | 1.11 | 1.00, 1.23 | 0.06 |
| CCI | 1.21 | 1.18, 1.24 | <0.001 |
| SAPSII | 1.02 | 1.02, 1.03 | <0.001 |
| SOFA | 1.07 | 1.05, 1.10 | <0.001 |
| Mechanical ventilation use |  |  |  |
| NO | 1.00 | Reference |  |
| YES | 0.89 | 0.78, 1.00 | 0.05 |
| Vasopressor use |  |  |  |
| NO | 1.00 | Reference |  |
| YES | 0.76 | 0.67, 0.86 | <0.001 |
| HF |  |  |  |
| NO | 1.00 | Reference |  |
| YES | 0.83 | 0.73, 0.94 | 0.005 |
| Renal |  |  |  |
| NO | 1.00 | Reference |  |
| YES | 0.74 | 0.63, 0.86 | <0.001 |
| Liver |  |  |  |
| NO | 1.00 | Reference |  |
| YES | 1.60 | 1.35, 1.91 | <0.001 |
| COPD |  |  |  |
| NO | 1.00 | Reference |  |
| YES | 1.57 | 1.37, 1.80 | <0.001 |
| CAD |  |  |  |
| NO | 1.00 | Reference |  |
| YES | 0.64 | 0.56, 0.72 | <0.001 |
| Stroke |  |  |  |
| NO | 1.00 | Reference |  |
| YES | 1.36 | 1.17, 1.58 | <0.001 |
| MAP | 1.00 | 1.00, 1.01 | 0.01 |
| Heart rate | 1.00 | 1.00, 1.01 | 0.009 |
| Temperature | 0.86 | 0.81, 0.91 | <0.001 |
| WBC | 1.00 | 1.00, 1.01 | 0.11 |
| Hemoglobin | 0.96 | 0.94, 0.99 | 0.002 |
| Platelet | 1.00 | 1.00, 1.00 | <0.001 |
| Sodium | 1.04 | 1.02, 1.06 | <0.001 |
| Potassium | 0.88 | 0.82, 0.93 | <0.001 |
| Bicarbonate | 0.97 | 0.96, 0.99 | 0.003 |
| Chloride | 0.95 | 0.93, 0.96 | <0.001 |
| BUN | 1.00 | 1.00, 1.01 | 0.006 |
| Lactate | 0.96 | 0.93, 1.0 | 0.02 |
| pH | 0.31 | 0.13, 0.70 | 0.005 |
| PO2 | 1.00 | 1.00, 1.00 | <0.001 |
| PCO2 | 0.99 | 0.98, 0.99 | <0.001 |
| Creatinine | 0.88 | 0.83, 0.92 | <0.001 |
| ^1^OR = Odds Ratio, CI = Confidence Interval | | | |

Supplementary Table 9. Survey-weighted generalised linear model adjusted with IPTW for 1-year mortality of cohort

| **Characteristic** | **OR**^1^ | **95% CI**^1^ | **p-value** |
| --- | --- | --- | --- |
| Group |  |  |  |
| Non-QTP | 1.00 | Reference |  |
| QTP | 1.34 | 1.15, 1.57 | <0.001 |
| Age | 1.01 | 1.00, 1.01 | 0.06 |
| Gender |  |  |  |
| Female | 1.00 | Reference |  |
| Male | 1.11 | 0.93, 1.31 | 0.25 |
| CCI | 1.21 | 1.16, 1.27 | <0.001 |
| SAPSII | 1.02 | 1.01, 1.03 | <0.001 |
| SOFA | 1.07 | 1.03, 1.11 | <0.001 |
| Mechanical ventilation use |  |  |  |
| NO | 1.00 | Reference |  |
| YES | 0.89 | 0.73, 1.08 | 0.23 |
| Vasopressor use |  |  |  |
| NO | 1.00 | Reference |  |
| YES | 0.76 | 0.62, 0.92 | 0.006 |
| HF |  |  |  |
| NO | 1.00 | Reference |  |
| YES | 0.83 | 0.68, 1.01 | 0.07 |
| Renal |  |  |  |
| NO | 1.00 | Reference |  |
| YES | 0.74 | 0.57, 0.95 | 0.02 |
| Liver |  |  |  |
| NO | 1.00 | Reference |  |
| YES | 1.60 | 1.23, 2.09 | <0.001 |
| COPD |  |  |  |
| NO | 1.00 | Reference |  |
| YES | 1.57 | 1.25, 1.98 | <0.001 |
| CAD |  |  |  |
| NO | 1.00 | Reference |  |
| YES | 0.64 | 0.52, 0.78 | <0.001 |
| Stroke |  |  |  |
| NO | 1.00 | Reference |  |
| YES | 1.36 | 1.07, 1.73 | 0.01 |
| MAP | 1.00 | 1.00, 1.01 | 0.12 |
| Heart rate | 1.00 | 1.00, 1.01 | 0.12 |
| Temperature | 0.86 | 0.78, 0.95 | 0.003 |
| WBC | 1.00 | 1.00, 1.01 | 0.23 |
| Hemoglobin | 0.96 | 0.93, 1.00 | 0.06 |
| Platelet | 1.00 | 1.00, 1.00 | 0.002 |
| Sodium | 1.04 | 1.02, 1.07 | 0.001 |
| Potassium | 0.88 | 0.79, 0.97 | 0.01 |
| Bicarbonate | 0.97 | 0.95, 1.00 | 0.07 |
| Chloride | 0.95 | 0.93, 0.97 | <0.001 |
| BUN | 1.00 | 1.00, 1.01 | 0.08 |
| Lactate | 0.96 | 0.91, 1.02 | 0.18 |
| pH | 0.31 | 0.07, 1.34 | 0.12 |
| PO2 | 1.00 | 1.00, 1.00 | 0.004 |
| PCO2 | 0.99 | 0.97, 1.00 | 0.02 |
| Creatinine | 0.88 | 0.81, 0.95 | <0.001 |
| ^1^OR = Odds Ratio, CI = Confidence Interval | | | |

Supplementary Table 10. Multivariate Cox model adjusted with all covariates for 1-year mortality of original cohort

| **Characteristic** | **HR**^1^ | **95% CI**^1^ | **p-value** |
| --- | --- | --- | --- |
| Group |  |  |  |
| Non-QTP | 1.00 | Reference |  |
| QTP | 1.23 | 1.10, 1.38 | <0.001 |
| Age | 1.01 | 1.00, 1.01 | 0.02 |
| Gender |  |  |  |
| Female | 1.00 | Reference |  |
| Male | 1.04 | 0.93, 1.17 | 0.47 |
| CCI | 1.17 | 1.14, 1.20 | <0.001 |
| SAPSII | 1.02 | 1.01, 1.02 | <0.001 |
| SOFA | 1.06 | 1.04, 1.09 | <0.001 |
| Mechanical ventilation use |  |  |  |
| NO | 1.00 | Reference |  |
| YES | 0.84 | 0.73, 0.96 | 0.01 |
| Vasopressor use |  |  |  |
| NO | 1.00 | Reference |  |
| YES | 0.83 | 0.72, 0.95 | 0.007 |
| HF |  |  |  |
| NO | 1.00 | Reference |  |
| YES | 0.89 | 0.78, 1.03 | 0.12 |
| Renal |  |  |  |
| NO | 1.00 | Reference |  |
| YES | 0.74 | 0.63, 0.88 | <0.001 |
| Liver |  |  |  |
| NO | 1.00 | Reference |  |
| YES | 1.33 | 1.12, 1.58 | 0.001 |
| COPD |  |  |  |
| NO | 1.00 | Reference |  |
| YES | 1.23 | 1.06, 1.42 | 0.006 |
| CAD |  |  |  |
| NO | 1.00 | Reference |  |
| YES | 0.68 | 0.59, 0.79 | <0.001 |
| Stroke |  |  |  |
| NO | 1.00 | Reference |  |
| YES | 1.22 | 1.04, 1.43 | 0.01 |
| MAP | 1.00 | 1.00, 1.00 | 0.42 |
| Heart rate | 1.00 | 1.00, 1.01 | <0.001 |
| Temperature | 0.87 | 0.81, 0.92 | <0.001 |
| WBC | 1.00 | 1.00, 1.01 | 0.27 |
| Hemoglobin | 0.98 | 0.95, 1.00 | 0.06 |
| Platelet | 1.00 | 1.00, 1.00 | 0.001 |
| Sodium | 1.03 | 1.02, 1.05 | <0.001 |
| Potassium | 0.92 | 0.85, 0.99 | 0.02 |
| Bicarbonate | 0.99 | 0.97, 1.01 | 0.22 |
| Chloride | 0.96 | 0.95, 0.97 | <0.001 |
| BUN | 1.00 | 1.00, 1.01 | 0.01 |
| Lactate | 0.97 | 0.94, 1.00 | 0.1 |
| pH | 0.52 | 0.22, 1.24 | 0.14 |
| PO2 | 1.00 | 1.00, 1.00 | <0.001 |
| PCO2 | 0.99 | 0.99, 1.00 | 0.03 |
| Creatinine | 0.92 | 0.87, 0.97 | 0.002 |
| ^1^HR = Hazard Ratio, CI = Confidence Interval | | | |

Supplementary Table 11. Multivariate Cox model adjusted with IPTW for 1-year mortality of cohort

| **Characteristic** | **HR**^1^ | **95% CI**^1^ | **p-value** |
| --- | --- | --- | --- |
| Group |  |  |  |
| Non-QTP | 1.00 | Reference |  |
| QTP | 1.22 | 1.08, 1.37 | 0.001 |
| Age | 1.01 | 1.00, 1.01 | 0.01 |
| Gender |  |  |  |
| Female | 1.00 | Reference |  |
| Male | 1.08 | 0.94, 1.23 | 0.27 |
| CCI | 1.15 | 1.11, 1.18 | <0.001 |
| SAPSII | 1.01 | 1.01, 1.02 | <0.001 |
| SOFA | 1.07 | 1.04, 1.10 | <0.001 |
| Mechanical ventilation use |  |  |  |
| NO | 1.00 | Reference |  |
| YES | 0.91 | 0.78, 1.06 | 0.22 |
| Vasopressor use |  |  |  |
| NO | 1.00 | Reference |  |
| YES | 0.80 | 0.69, 0.94 | 0.005 |
| HF |  |  |  |
| NO | 1.00 | Reference |  |
| YES | 0.89 | 0.76, 1.03 | 0.11 |
| Renal |  |  |  |
| NO | 1.00 | Reference |  |
| YES | 0.79 | 0.66, 0.95 | 0.01 |
| Liver |  |  |  |
| NO | 1.00 | Reference |  |
| YES | 1.39 | 1.15, 1.68 | <0.001 |
| COPD |  |  |  |
| NO | 1.00 | Reference |  |
| YES | 1.37 | 1.17, 1.62 | <0.001 |
| CAD |  |  |  |
| NO | 1.00 | Reference |  |
| YES | 0.71 | 0.60, 0.84 | <0.001 |
| Stroke |  |  |  |
| NO | 1.00 | Reference |  |
| YES | 1.26 | 1.06, 1.50 | 0.01 |
| MAP | 1.00 | 1.00, 1.01 | 0.16 |
| Heart rate | 1.00 | 1.00, 1.01 | 0.02 |
| Temperature | 0.89 | 0.83, 0.96 | 0.003 |
| WBC | 1.00 | 1.00, 1.00 | 0.27 |
| Hemoglobin | 0.99 | 0.96, 1.01 | 0.32 |
| Platelet | 1.00 | 1.00, 1.00 | 0.002 |
| Sodium | 1.03 | 1.01, 1.05 | 0.004 |
| Potassium | 0.92 | 0.84, 0.99 | 0.03 |
| Bicarbonate | 0.98 | 0.96, 1.00 | 0.04 |
| Chloride | 0.96 | 0.95, 0.98 | <0.001 |
| BUN | 1.00 | 1.00, 1.01 | 0.02 |
| Lactate | 0.97 | 0.93, 1.01 | 0.13 |
| pH | 0.56 | 0.19, 1.65 | 0.29 |
| PO2 | 1.00 | 1.00, 1.00 | 0.004 |
| PCO2 | 0.99 | 0.98, 1.00 | 0.06 |
| Creatinine | 0.90 | 0.85, 0.96 | <0.001 |
| ^1^HR = Hazard Ratio, CI = Confidence Interval | | | |

Supplementary Table 12. Sensitivity analysis of the association between Framingham-defined QT Prolongation and 28-day mortality

| **Method** | **Adjusted Ratios** | **CI** |  | ***p* value** |
| --- | --- | --- | --- | --- |
|  |  | **2.5%** | **97.5%** |  |
| Multivariate logistic model adjusted with all covariates (OR) | 1.36 | 1.03 | 1.77 | 0.03 |
| Multivariate logistic model adjusted with IPTW (OR) | 1.49 | 1.32 | 1.67 | <0.001 |
| Survey-weighted generalized linear model adjusted with IPTW (OR) | 1.49 | 1.08 | 2.04 | 0.01 |
| Multivariate Cox model adjusted with all covariates (HR) | 1.22 | 0.97 | 1.52 | 0.08 |
| Multivariate Cox model adjusted with IPTW (HR) | 1.39 | 1.07 | 1.81 | 0.01 |

Supplementary Table 13. Sensitivity analysis of the association between Framingham-defined QT Prolongation and 1-year mortality

| **Method** | **Adjusted Ratios** | **CI** |  | ***p* value** |
| --- | --- | --- | --- | --- |
|  |  | **2.5%** | **97.5%** |  |
| Multivariate logistic model adjusted with all covariates (OR) | 1.54 | 1.22 | 1.93 | <0.001 |
| Multivariate logistic model adjusted with IPTW (OR) | 1.55 | 1.40 | 1.71 | <0.001 |
| Survey-weighted generalized linear model adjusted with IPTW (OR) | 1.55 | 1.18 | 2.04 | 0.002 |
| Multivariate Cox model adjusted with all covariates (HR) | 1.28 | 1.08 | 1.51 | 0.004 |
| Multivariate Cox model adjusted with IPTW (HR) | 1.38 | 1.13 | 1.69 | 0.002 |
